# Outcomes of Elective Shoulder Arthroplasty: A Single-Center Service Evaluation

**DOI:** 10.7759/cureus.94337

**Published:** 2025-10-11

**Authors:** Thet Paing Oo, Bishoy Toma, Hassan Imtiaz, Carlos Argerich, Georgios Kouklidis, May Oo Cho, Senthil Muthian

**Affiliations:** 1 Trauma and Orthopedics, University Hospitals Dorset NHS Foundation Trust, Poole, GBR; 2 Orthopedics, University Hospitals Dorset NHS Foundation Trust, Poole, GBR; 3 Stroke, Norfolk and Norwich University Hospitals NHS Foundation, Norwich, GBR; 4 Trauma and Orthopedics, Poole General Hospital, Poole, GBR

**Keywords:** asa classification, audit, elective orthopedic surgery, length of stay, reverse shoulder arthroplasty, shoulder arthroplasty

## Abstract

Introduction

Elective shoulder arthroplasty is increasingly performed in elderly patients with degenerative shoulder pathology. Optimizing perioperative care is essential to minimize complications and length of stay (LOS).

Methods

A retrospective audit was conducted of all elective shoulder arthroplasty procedures performed at our center between January 2024 and April 2025. Revision surgeries (n=11) and trauma cases (n=1) were excluded. Data on patient demographics, American Society of Anesthesiologists (ASA) classification, surgical indications, procedure type, LOS, readmissions, and surgical timing were analyzed.

Results

A total of 72 patients were evaluated. The majority of patients (n=51, 70.8%) were aged 71-90 years. Reverse shoulder arthroplasty was performed in 57 (79.2%) cases. Most patients were ASA II (n=48, 66.7%), with 33.3% (n=24) having an ASA score of III. Degenerative joint disease (arthritis with or without cuff tear) was the most common indication (n=52, 72.2%). Median LOS was one to three days; 36.1% (n=26) were discharged after a one-day stay. Complications (anemia, hyponatremia, hypotension) and social factors were the main causes of extended stay. Readmissions were rare (n=1, 1.4%).

Conclusion

Elective shoulder arthroplasty can be safely delivered with short LOS and low readmission rates. Enhanced preoperative assessment, early physiotherapy and imaging, and streamlined discharge planning may further improve efficiency and bed utilization.

## Introduction

Shoulder arthroplasty is a well-established surgical option for degenerative glenohumeral arthritis and cuff tear arthropathy, particularly in elderly patients [1,2]. Reverse shoulder arthroplasty (RSA) has largely supplanted anatomic total shoulder arthroplasty (TSA) in this group due to improved functional outcomes and lower revision risk [3,4]. In the United Kingdom and internationally, procedural volumes have steadily increased over the past decade [5,6].

While outcomes are generally excellent, the growing demand creates pressure to reduce inpatient length of stay (LOS) and associated healthcare costs without compromising safety [7]. Enhanced recovery pathways (ERPs) have been widely adopted in arthroplasty of the hip and knee, showing reduced LOS and complications [8], and are increasingly being applied to shoulder arthroplasty [9,10].

Despite this, there is limited literature evaluating real-world LOS, complications, and readmissions in contemporary elective shoulder arthroplasty cohorts. Understanding these metrics is crucial to optimizing perioperative planning, especially in elderly, comorbid patients who often require coordinated discharge support [11,12]. This service evaluation project examines patient demographics, LOS, complications, and readmissions following elective shoulder arthroplasty at our center. This study aimed to identify trends in shoulder arthroplasty surgery choices, compare their outcomes and length of hospital stay, and align the findings with current literature.

## Materials and methods

A retrospective study of all shoulder arthroplasty cases performed between January 2024 and April 2025 was conducted at Poole General Hospital, United Kingdom. Cases were identified from operative logs. Patients over 18 years old undergoing elective shoulder arthroplasty were included in the study. Those undergoing revision surgeries (n=11) and trauma cases (n=1) were excluded, leaving a total of 72 elective procedures for final analysis. Data were collected from electronic health records and Picture Archiving and Communication System (PACS). Measured outcomes included age, sex, ASA classification, surgical indication, procedure type (RSA or anatomic shoulder replacement), length of stay (LOS), readmissions within 90 days, and timing of surgery (morning vs. afternoon lists).

Data were collected and stored on the hospital intranet system on a Microsoft Excel spreadsheet (Redmond, WA: Microsoft Corp.). Patient identifiers were removed, and given that the data were stored on the hospital intranet system, this avoided any breach of confidentiality. Descriptive statistics were used to summarize data. As part of the service evaluation, formal ethics approval was not required [13].

## Results

Seventy-two patients met the inclusion criteria for the duration of the study. Mean age was 72.9 years (±9.8 years). Most patients were aged 71-90 years (70.8%), with the age distribution presented in Table 1. Sixteen of 72 patients (21.8%) underwent anatomic shoulder replacement, while the remaining 57 of 72 (79.2%) received reverse shoulder arthroplasty. The predominant ASA classification was grade 2 (n=48, 66.7%), followed by ASA grade 3 in 24 patients (33.3%). None of the patients had an ASA grade higher than 3. These results are presented in Table 2.

**Table 1:** Age distribution of study participants.

Age group (years)	Number of patients, n (%)
21-30	1 (1.4%)
31-40	0
41-50	3 (4.2%)
51-60	4 (5.5%)
61-70	13 (18%)
71-80	36 (50%)
81-90	15 (20.8%)
91+	0

**Table 2:** Procedure type and ASA grade of study participants. ASA: American Society of Anesthesiologists

Procedure type	Number of patients, n (%)
Anatomic shoulder replacement	15 (20.8%)
Reverse shoulder arthroplasty	57 (79.2%)
ASA grade	
I	0
II	48 (66.7%)
III	24 (33.3%)

Surgical indications predominantly included arthritis in 43/72 patients, accounting for 59.7% of shoulder arthroplasties. For the remaining patients, 16/72 (22.2%) had rotator cuff tears alone, 9/72 (12.5%) had arthritis with a rotator cuff tear, 3/72 (4.2%) had non-union of the humeral head, and 2/72 (2.8%) suffered from avascular necrosis of the humeral head. Out of all the 72 procedures, only one patient (1.4%) required a readmission within four months of surgery, due to aseptic loosening of the implant. Mean length of stay was 1.7 days (range: 1-3 days). This was distributed as follows: 26 of 72 patients (36%) were discharged the day after surgery (within 24 hours) following imaging and physiotherapy assessment, while 31 of 72 patients (43%) were discharged on day two or three postoperatively, with delays attributed to postponed physiotherapy assessments and the need for pain control optimization. Thirteen of 72 patients (18%) were discharged within a three to 10-day period, with the primary reasons being medical complications such as postoperative anemia, electrolyte imbalances, suboptimal pain control, or hypotension. Two of 72 patients (2.8%) remained in hospital for more than 10 days, both awaiting care packages prior to discharge. Surgical timing was evenly distributed between morning (n=39, 51.4%) and afternoon (n=35, 48.6%) operating lists. Afternoon cases more often required next-day physiotherapy and radiographs, contributing to delayed discharge. Surgical indications, timing, and mean LOS are presented in Table 3.

**Table 3:** Surgical indications, timing, and mean length of stay (LOS).

Surgical indication	Number of patients, n (%)
Rotator cuff tear alone	16
Arthritis	43
Arthritis + cuff tear	9
Avascular necrosis (AVN)	1
AVN + cuff tear	1
Non-union humeral head	3
Surgical timing	
Morning	37 (51.4%)
Afternoon	35 (48.6%)
Mean LOS (days)	1.7 days (range: 0-13 days)
Within 24 hours	26 (36%)
1-3 days	31 (43%)
3-10 days	13 (18%)
>10 days	2 (2.8%)

## Discussion

This single-center audit demonstrates that elective shoulder arthroplasty can be delivered with a short median length of stay (LOS: 1-3 days), low complication-related delays, and very low readmission rates (1.4%) in a predominantly elderly and comorbid cohort. Notably, more than one-third of patients (36.1%) were discharged within 24 hours, reflecting the increasing feasibility of early discharge in shoulder arthroplasty when perioperative care is optimized.

The LOS observed in our series is consistent with reports from recent multicenter studies, which describe median stays of one to two days following elective shoulder arthroplasty [14,15]. The shift toward shorter admissions is driven by enhanced recovery after surgery (ERAS) principles, which have been shown to reduce LOS and complications without increasing readmissions [9,10]. Our 1.4% readmission rate compares favorably with reported rates of 1-3% in the wider literature [16-18].

Reverse shoulder arthroplasty (RSA) accounted for 79.2% of procedures, reflecting international trends toward RSA dominance in elderly patients with cuff-deficient shoulders [3,4,16]. Comparative studies demonstrate that RSA provides superior functional outcomes and pain relief in this population compared with anatomic total shoulder arthroplasty (TSA), with lower revision risk in the medium term [4,16]. TSA remains the gold standard for younger patients with intact rotator cuffs, but the expansion of RSA indications has been well-documented in registry analyses [5,6].

Most complications in our cohort were minor medical issues, including hyponatremia (n=4), postoperative anemia (n=2), hypotension (n=2), and isolated cases of cardiac arrest and uncontrolled pain. These complications are consistent with those described in other arthroplasty series and reflect the vulnerability of elderly surgical patients to metabolic and cardiovascular derangements [19]. Importantly, only one patient (1.4%) required readmission, due to aseptic loosening, which is lower than registry-reported revision rates at short-term follow-up [17].

Social factors also contributed to extended LOS, with two patients delayed >10 days while awaiting care packages. Previous studies highlight that discharge planning and community resource availability are crucial determinants of LOS in orthopedic surgery [11,20]. Our findings reinforce the importance of early multidisciplinary discharge planning to avoid unnecessary bed occupancy. Afternoon surgical timing was associated with more frequent next-day physiotherapy and radiographic assessments, delaying discharge compared with morning cases. This aligns with prior work showing that same-day rehabilitation access is a key determinant of 24 h discharge rates [20,21].

Although our audit was not designed as a survivorship study, our low early revision/readmission rates are in line with national registry reports. Data from joint replacement registries report 10-year survivorship of 88-96% for both TSA and RSA, depending on indication and patient age [5,16]. Younger age and revision surgery remain strong predictors of implant failure [2,6]. Our early aseptic loosening case highlights the need for ongoing follow-up and longer-term analysis to assess implant performance.

These findings support continued development of ERAS pathways in shoulder arthroplasty. Early mobilization, standardized imaging access, and streamlined physiotherapy services can facilitate same-day or next-day discharge in carefully selected patients. Proactive preoperative optimization of hemoglobin and electrolytes may further reduce metabolic complications and avoid delays. Importantly, our results suggest that early discharge is safe and does not increase readmission risk when perioperative protocols and follow-up are robust.

This study has several limitations. First, it is a retrospective audit from a single center, limiting generalizability. Second, functional outcome measures such as Constant or ASES scores were not captured, restricting comparison with functional outcomes reported in other series. Third, follow-up was limited to 90-day readmissions; longer-term complications and survivorship could not be assessed. Fourth, the cohort was relatively small (n=72), which may have underestimated the incidence of rare complications. Finally, the predominance of elderly patients undergoing RSA reflects our local demographics and may not be representative of younger populations where TSA remains more common. Further multicenter studies with larger sample sizes and more comparable groups could allow for statistical analysis and generalizability of results.

## Conclusions

Elective shoulder arthroplasty at our center was associated with a short LOS (median: 1-3 days), low complication-related delays, and very low readmission rates (1.4%). RSA was the dominant procedure, reflecting the ageing and comorbid patient cohort. Targeted preoperative optimization, enhanced recovery protocols, and improved discharge coordination may further streamline care and support earlier discharge without compromising safety.
